# Legal and State Medicine

**Published:** 1899-09

**Authors:** William Hobson Heath

**Affiliations:** Buffalo, N. Y.


### LEGAL AND STATE MEDICINE.

Conducted by WILLIAM HOBSON HEATH, M. D., Buffalo, N. Y.

IN NO branch of the science of medicine are medical men so deficient, in none so poorly trained, as in medical jurisprudence, particularly in this country. The amount of knowledge which concerns the legal and medical professions in common, has never been so extensive or important, nor the necessity for the application of medical knowledge to elucidate legal questions, or determine justice, become so frequent and necessary.

The possibilities, in view of these facts, the present state of medical science, the physician himself, in relation to legal affairs, justifies the Journal devoting a part of its energy in this field.

Matter presented to the busy practitioner will be practical, concise and absolutely up-to-date. The relationship of medico-legal, and state interests will also permit the presentation of matter and ideas upon this subject which will be made interesting as they are valuable.

### HOSPITAL LIABILITY.

The question of hospital liability to patients in event of certain conditions arising, is one of the utmost importance, and while the subject is yet in a transitional state one phase of it has arisen and been passed upon by no less authority than the Appellate Division of the Supreme Court, State of New York, viz.: Ward vs. St. Vincent's Hospital. The case in question concerns the hospital's liability for negligence on the part of its servants. Briefly, Miss Ward, about to be operated upon by her private surgeon (one of the visiting surgeons on the staff), engaged a private $25.00 per week room at the hospital, with the usual services and advantages that are incident to or are given private room pay-patients, and additionally in view of her peculiar dread of anesthetics, contracted with the hospital for an extra special, skilled, trained nurse, at the rate of $3.00 per day; total expense, $46.00. While *unconscious* she was carried from the operating to her own room, and put into a bed, from which the nurse had neglected to remove an unprotected rubber hot-water bag. As a result of laying upon this bag, her leg was burned, so seriously as to prolong her recovery, increase her suffering and leave her permanently injured—making a great change in her manner of life, which was formerly one of great physical activity—riding, golf, outdoor, etc.

Suit was brought and went against her in the Supreme Court, Judge Cohen dismissing the case on the following grounds: (1) That it was one of "tort" not contract; (2) that the hospital was liable only for the original selection of its servants—unless unfitness was shown since; (3) that it was a charitable institution, not run to make money, declare dividends, etc., the profits from pay-patients going to support the charity ones; (4) that placing the liability upon them as such it would restrict their efforts to suffering humanity, liability acting as a deterring effect; (5) that it is necessary occasionally for the one to suffer for the many.

On being carried to the Appellate Division, all the justices concurred in granting a new trial. Its decision drew a distinction between the public ward and private room departments. The latter, in this case, entered into a distinct agreement and contract to do certain things which they did not do in full, as it appeared that instead of supplying skilled, fully trained nurse, they substituted a person who had only had nine months out of the two years' training course; that had a distinct *trained*, not *training* nurse, been placed on duty, as called for in the contract, the accident most likely would not have occurred.

The decision completely ignores the lower court's view of the matter and is most interesting, establishing, as it does, a precedent for some time to come.

The question of the hospital's liability in cases of, or cases of alleged malpractice to this extent being now established and a precedent made, it naturally suggests the question of liability in the case of the attending surgeons or the attending staff. The relationship of these two bodies are peculiar and their mutual interdependence and benefit arising to each is often incidentally lost sight of.

The hospital holds the confidence and patronage of the public largely from the skill, ability and reputation of its attending unpaid officers, and is the sole recipient of pecuniary advantages. The services of the attending staff are frequently so great that it is a matter of surprise to many that it receives no compensation therefor; that the only unpaid for service is the most important and vital. The staff, on the other hand, though receiving no money return, is undoubtedly rewarded by the reputation the holding of such position gives, the public presupposing high ability, by the opportunity it affords for observation, study, and the rapid acquirement of skill and experience, as an indirect result, and practice and emolument.

From analogy on the Ward case, it would appear that a distinction is to be drawn in the two departments of a hospital when they exist (in private room practice the liability is much simplified by the fact that the patient generally arranges with the surgeon direct and thus relieves the institution). In ward practice, however, the conditions are different, as is well known. Here the hospital could be held on the ground of principal and agent, lack of money consideration being no evasion, nor the advantage of experience, etc., which are benefits merely incident to the duty voluntarily assumed. On the other hand, action against the surgeon alone is warranted on the ground that he alone is in control of and has direct and immediate control of his action. Joint action against the hospital and the surgeon is justified on the ground of "joint tort feasors." The distinct settlement of this question will not be without gain to all concerned, particularly the public, inasmuch as it will determine great care in the selection of the attending staff, giving good grounds for the elimination of religious, political, or other influence in the appointing, and making the surgeon more careful if possible, deterring unjustifiable operations and other practises open to criticism.

Features of interest brought out in this case is the differentiation of the liability or degree of liability in the private room pay department of charitable institutions. In it the liability of the hospital is established, whereas in the charitable side it would be governed by a different construction, viz. : that where a corporation is conducting a charity with funds intended for that purpose it is not liable for the "torts" of its agents or servants, as it would be against all law and equity to take funds contributed for charitable purposes to compensate for injuries inflicted or occasioned by neglect. This limitation of liability is further justified that successful financial management is applied for the benefit of suffering humanity and not to profit-bearing.

A number of points bearing upon this question will be taken up in the near future to the extent of presenting the case and status by existing decisions, not at this writing obtainable.

---

PREVENTION OF CHOLERA INFANTUM.

HORATIO C. WOOD affirms (*International Clinics*) that this disease is often nothing more than heat exhaustion with vasomotor paralysis and a leakage of serum from the bloodvessels into the intestines. The prompt reduction of high temperature is indicated if the case is seen early before collapse, but when this has taken place the application of heat and the internal use of stimulants must be resorted to. Prophylaxis, says the writer, is as easy as for ordinary sunstroke. "Keep the child in a cool, shaded place, with plenty of air and as little clothing as possible." If the weather is very warm, bathing the child twice a day in front of an open window will help to keep the temperature normal. In young infants who appeared perfectly well, he has frequently found a temperature of 102° or 103° after several successive days of hot weather.—*Denver Med. Times.*

---

DR. WALTER B. DORSETT, professor of obstetrics in Beaumont Hospital College says: "Although I once was enthused with the belief that it was a feasible undertaking to treat successfully uterine fibroids by the ligation of the uterine arteries per vaginam, I now know in the light of subsequent experience, it is not worth the trial, and I consider the ligation of the broad ligament, en masse, which means a thorough obliteration of arteries, veins, nerves and the like, contained in the broad ligament, as practised by Martin, as unworthy of a place in medical literature."—*Amer. Jour. Surg. and Gyne.*, August, 1899.

---

ACCORDING to the *Medical Record*, under the Spanish rule, in order to practice legally in Cuba, it was necessary to hold a diploma of the University of Havana or of some University in Spain, viséd by the proper authority of the district. Those holding foreign diplomas were obliged to pass an examination or to obtain a temporary license. Since the expulsion of the Spaniards, the *Archives de la Policlinica* complains, many foreigners have come to the island and begun practice without let or hindrance. Our contemporary naturally holds that such an abuse should be stopped by the authorities now governing the country.—*Medical Age.*

